# Twenty-year follow up of the TIPS study

**DOI:** 10.1192/j.eurpsy.2025.133

**Published:** 2025-08-26

**Authors:** W. T. V. Hegelstad, M. Weibell

**Affiliations:** 1 TIPS Centre for Clinical Research in Psychosis, Stavanger University Hospital; 2 Faculty of Social Sciences, University of Stavanger, Stavanger; 3 Medical Faculty, University of Bergen, Bergen, Norway

## Abstract

**Background:**

Comparing long-term outcomes in psychosis can be challenging due to widely differing definitions, measures, and populations. The remission working group led by Andreasen et al. suggested remission criteria based on positive symptoms and duration. However, there is no internationally agreed definition of functional recovery. The TIPS study is one of very few with very long-term follow-up, and this presents a unique opportunity both to investigate outcomes as well as use findings to develop valid definitions for the future.

**Objective:**

In this longitudinal study, we explore remission patterns and functional status with regards to social contacts, living situation and employment status over 20 years.

**Methods:**

A representative sample originally consisting of 201 first episode psychosis patients from two Norwegian well defined catchment areas in the Scandinavian TIPS study have been followed for more than 20 years with symptom and functional measures. Assessments have taken place at inclusion, one, two, five, ten and twenty years. At the 20-year follow-up, 43% of living participants were retained; 15% had died.

**Results:**

Data analysis is in progress, and symptoms and function results will be presented.

**Disclosure of Interest:**

None Declared

